# The emergence of SARS-CoV-2 variants of concern is driven by acceleration of the substitution rate

**DOI:** 10.1093/molbev/msac013

**Published:** 2022-01-17

**Authors:** John H Tay, Ashleigh F Porter, Wytamma Wirth, Sebastian Duchene

**Affiliations:** Peter Doherty Institute for Infection and Immunity, University of Melbourne, Melbourne, Australia; Peter Doherty Institute for Infection and Immunity, University of Melbourne, Melbourne, Australia; Peter Doherty Institute for Infection and Immunity, University of Melbourne, Melbourne, Australia; Peter Doherty Institute for Infection and Immunity, University of Melbourne, Melbourne, Australia

**Keywords:** SARS-CoV-2 molecular evolution, variants of concern, molecular clock, Bayesian model selection

## Abstract

The ongoing SARS-CoV-2 pandemic has seen an unprecedented amount of rapidly generated genome data. These data have revealed the emergence of lineages with mutations associated to transmissibility and antigenicity, known as variants of concern (VOCs). A striking aspect of VOCs is that many of them involve an unusually large number of defining mutations. Current phylogenetic estimates of the substitution rate of SARS-CoV-2 suggest that its genome accrues around 2 mutations per month. However, VOCs can have 15 or more defining mutations and it is hypothesized that they emerged over the course of a few months, implying that they must have evolved faster for a period of time. We analysed genome sequence data from the GISAID database to assess whether the emergence of VOCs can be attributed to changes in the substitution rate of the virus and whether this pattern can be detected at a phylogenetic level using genome data. We fit a range of molecular clock models and assessed their statistical performance. Our analyses indicate that the emergence of VOCs is driven by an episodic increase in the substitution rate of around 4-fold the background phylogenetic rate estimate that may have lasted several weeks or months. These results underscore the importance of monitoring the molecular evolution of the virus as a means of understanding the circumstances under which VOCs may emerge.

## Introduction

### The molecular clock of SARS-CoV-2

Genome sequence data of viruses have been extensively used to track the evolution and spread of these pathogens. The ongoing SARS-CoV-2 pandemic has seen an unprecedented number of genomes generated that have been used to gain rapid insight to understand epidemiological spread (Dellicour *et al.*, 2021), identify the time of origin (Pekar *et al.*, 2021), and track mutations of functional importance. Most concerning mutations occur in the spike protein and may increase transmissibility (Kraemer *et al.*, 2021), or disease severity (Harvey *et al.*, 2021), although vaccines are likely still effective against them (Dearlove *et al.*, 2020). Such lineages are known as variants of concern (VOCs) and they are characterized at a genomic level by a number of fixed mutations in the S1 subunit of the spike protein, the most common of which are mutations N501Y and D614G (Eurosurveillance, 2021), with the the latter presenting evidence increased transmissibilty and favoured by selection (Martin *et al.*, 2021; Volz *et al.*, 2021). For a lineage to be formally denominated as a VOC there must be evidence of an impact in transmissibility, virulence, and/or immunity (Mascola *et al.*, 2021).

SARS-CoV-2 lineages are classified using a dynamic nomenclature system, known as PANGO (Rambaut *et al.*, 2020). Recently the World Health Organisation assigned VOCs letters of the greek alphabet (Konings *et al.*, 2021). In October 2021 the United States CDC recognizes four VOCs; Alpha (PANGO lineage B.1.1.7) first identified in the UK, Beta (PANGO lineage B.1.351) first identified in South Africa, Gamma (PANGO lineage P.1) first identified in Brazil, and Delta (PANGO lineage B.1.617.2) first identified in India (CDC, 2021). As of December 2021 a new VOC has been detected and has rapidly spread globally (Viana *et al.*, 2021), Omicron (PANGO lineages BA.1 and BA.2), not included in this study.

The mechanisms under which VOCs have emerged is not entirely clear, but their defining mutations are well characterized and their fixation has been attributed to the action of natural selection (Martin *et al.*, 2021). Variant Alpha has 14 protein-altering mutations and three deletions, with eight of these being in the spike protein. One of the deletions ΔH69/ΔV70 enhances infectivity in vitro and has been detected in immunocompromised patients where immune escape occurred (Kemp *et al.*, 2021; Plante *et al.*, 2021). Variant Beta has nine protein-altering mutations with five altering the receptor binding domain. (Tegally *et al.*, 2021). Variant Gamma has 17 mutations, with 10 found in the spike protein and including N501Y and E484K (Faria *et al.*, 2021). Alpha, Beta and Gamma share several important mutations, including N501Y and E404K, which likely enhance affinity to human the ACE2 receptor (Nelson *et al.*, 2021). Variant Delta is characterized by 7 mutations in the spike protein, several of which have been associated with altered immune response and increased viral replication, viral load, and likely leading to increased viral fitness (CDC, 2021).

The sheer number of mutations observed in these four VOCs is much higher than what would be expected under phylogenetic estimates of the nucleotide evolutionary rate of SARS-CoV-2, which range from around $7\times10^{-4}$ to $1.1\times10^{-3}$ subs/site/year (Duchene *et al.*, 2020; Ghafari *et al.*, 2021), meaning that only about 2 mutations along the genome would accumulate per month along a lineage. In these circumstances, the 14 mutations in Alpha would require a period of at least six months, a time that is inconsistent with its first detection in September 2020, because it would have had to evolve from around March 2020

with most defining mutations undetected for many months.

We investigated whether the emergence of VOCs is associated with an increase in the evolutionary rate that can be detected using phylogenetic analyses of genome data and in the absence of dense intrahost or transmission chain sampling. The term 'evolutionary rate' refers to the amount of molecular change that can be measured using a phylogenetic method and is thus the result of the instantaneous mutation rate and the substitution rate (i.e. the rate at which such mutations become fixed) (Ho *et al.*, 2011). The latter is largely determined by the action of natural selection, which is a probable cause for the large number of mutations in VOCs (Martin *et al.*, 2021). Thus, here we use the term 'substitution rate', which reflects our estimates more closely. We analysed publicly available nucleotide sequence data from GISAID (Elbe and Buckland-Merrett, 2017; Shu and McCauley, 2017) under a range of molecular clock models that describe the substitution rate along branches in phylogenetic trees, shown in the Supplementary material. We consider each model as a hypothesis for which we can assess statistical support using Bayesian model selection techniques. Critically, our analyses do not intend to detect signatures of natural selection, nor to identify genomic regions with higher mutation rates, which have been described elsewhere (Abdool Karim and de Oliveira, 2021; Harvey *et al.*, 2021; Martin *et al.*, 2021). Instead, our framework serves to characterise the main patterns of substitution rate variation in the genome of the virus that underpin the emergence of VOCs.

The simplest molecular clock model is known as the strict molecular clock (SC; Zuckerkandl, 1962; Zuckerkandl and Pauling, 1965) that posits a single substitution rate for all branches in a phylogenetic tree, and thus serves as a 'null' model. A more complex model is the uncorrelated relaxed clock that assumes that branch rates are independent and identically distributed draws from a statistical distribution (Drummond *et al.*, 2006), for which we considered either a lognormal or a $\Gamma$ distribution (UCLN and UCG, respectively). We also considered a range of fixed local clock models (FLC; Yoder and Yang, 2000). These models require an *a priori* definition of a set of 'background' branches and a set of branches with different rates, known as 'foreground'. For example, foreground branches can be defined based on some biological expectation (e.g. Worobey *et al.*, 2014) and represent a formal evolutionary hypothesis. The substitution rate is constant for a given group of branches, although there exist approaches where branches can be assigned a set of relaxed molecular clocks (Fourment and Darling, 2018). These models differ in their number of parameters and biological assumptions (Supplementary material Table S1; reviewed in Bromham *et al.*, 2018 and Ho and Duchêne, 2014).

We specified six configurations of the FLC model, where the substitution rate could vary within VOC clades (FLC clades model in Supplementary material Figure S1) or along the stem (FLC stems+clades), only at stem branches (FLC stems), or where these rates could be shared among all VOCs (FLC shared stems, FLC shared clades and FLC shared clades+stems in Supplementary material Figure S1).

Models in which the rate only changes along the stem branches of VOCs represent a situation where the substitution rate may increase for a short period of time before returning to the background rate. In contrast, models where the clade also undergoes a rate change would imply that VOCs have a rate that is statistically different from the background.

An alternative approach to the FLC is the random local clock (RLC; Drummond and Suchard, 2010). The substitution rate can change at particular nodes in the tree and the location of such changes and actual rates are inferred. The RLC is a general form of all local clock models, where the simplest form is the strict clock, as a case of no rate changes (Bromham *et al.*, 2018; Ho and Duchêne, 2014).

### Bayesian hypothesis testing

We conducted Bayesian model testing by calculating the log marginal likelihood, a measure of statistical fit, and ranking the models accordingly. The difference in log marginal likelihoods between two models is known as the log Bayes factor (Sinsheimer *et al.*, 1996) and measures the relative support for two models given the data. In general, a log Bayes factor of at least 1.1 is considered as 'substantial evidence' in favour of a model, with 2.3 being 'strong' and 4.6 'decisive' (Kass and Raftery, 1995). We considered two marginal likelihood estimators, path sampling and stepping-stone sampling (Gelman and Meng, 1998; Lartillot and Philippe, 2006; Xie *et al.*, 2011).

## Results

### Model selection

The FLC shared stems model had the highest statistical fit, with a log Bayes factor of at least 1.92 compared to the next best-fitting model (2.30 with path-sampling and 1.92 with stepping-stone sampling; Table 1). The next model with highest mean log marginal likelihood was the UCG, followed by the FLC stems and UCLN. Note, however, that there is some overlap in replicate log marginal likelihoods for these four models (Fig 1). The two top FLC models assume that the stem branches of VOC have a rate that differs from the background and they only differ in that the FLC stems model allows each VOC stem branch to have its own rate.

The uncorrelated relaxed clocks had very similar performance, although both had at least 'substantial evidence' against them with respect to the FLC shared stems model (i.e. log Bayes factors > -1.1). The log Bayes factors for the remaining models were at least -13, implying

**Table 1.** Model selection results for complete SARS-CoV-2 genomes. Mean estimates of log marginal likelihoods using path sampling and stepping-stone (ps logML and ss logML, respectively), over 10 replicates. log Bayes factors (BF) are shown for the best-fitting model, relative to all others (larger numbers mean lower statistical fit), and thus they are 0.0 for the top model.

| Model | ps logML | ss logML | ps rank | ss rank | ps BF | ss BF |
|---|---|---|---|---|---|---|
| FLC shared stems | -55430.85 | -55431.49 | 1 | 1 | 0 | 0 |
| UCG | -55433.15 | -55433.41 | 2 | 2 | -2.30 | -1.92 |
| FLC stems | -55434.05 | -55434.51 | 3 | 3 | -3.2 | -3.02 |
| UCLN | -55435.83 | -55435.81 | 4 | 4 | -4.98 | -4.32 |
| SC | -55444.05 | -55444.59 | 5 | 5 | -13.20 | -13.10 |
| FLC shared clades+stems | -55444.77 | -55445.31 | 6 | 6 | -13.92 | -13.82 |
| FLC shared clades | -55449.82 | -55450.29 | 7 | 7 | -18.97 | -18.80 |
| FLC clades+stems | -55453.62 | -55454.09 | 8 | 8 | -22.77 | -22.60 |
| FLC | -55458.51 | -55459.06 | 9 | 9 | -27.66 | -27.57 |

'decisive' evidence against them, relative to the FLC shared stems.

Interestingly, FLC models where VOC clades were defined as foreground had decisively lower statistical performance than those where only stem branches were labelled as foreground (Table 1, 1). In fact, even the SC model, which is generally considered unrealistic for empirical data, had a log Bayes factor of at least 4 with respect to FLC shared clades and the FLC clades+stems Table 1.

## Rates of evolution of variants of concern

The FLC shared stems model had a mean background substitution rate of $0.58\times10^{-3}$ subs/site/year (95% CI: 0.51 - $0.65\times10^{-3}$), while that for the VOC stems was $2.45\times10^{-3}$ subs/site/year (95% CI: 1.15 - $4.72\times10^{-3}$). The corresponding mean values in units of substitutions per month across the entire genome (subs/month) are 1.24 for the background and 6.11 for the foreground. As such, the VOC stems rate was around 4 fold higher than the background (mean 4.25, 95% CI: 2.61 - 8.19) (Fig 2).

Although the FLC stems model that assigned each VOC stem branch a different rate had very high uncertainty, it also suggested much higher rates for these branches. The mean background rate under this model was $0.55\times10^{-3}$ subs/site/year (95% CI: 0.49 - $0.62\times10^{-3}$). The corresponding values for VOC were $8.47\times10^{-3}$ subs/site/year (95% CI: 1.93 - $82.37\times10^{-3}$) for Alpha, $1.71\times10^{-3}$ (95% CI: 0.34 - $33.20\times10^{-3}$) for Beta, $2.76\times10^{-3}$ (95% CI: 1.21 - $13.23\times10^{-3}$) for Gamma, and $1.54\times10^{-3}$ (95% CI: 0.62 - $7.35\times10^{-3}$) for Delta. Clearly, these estimates were several fold higher than that of the background branches, and in spite of their high uncertainty least 0.90

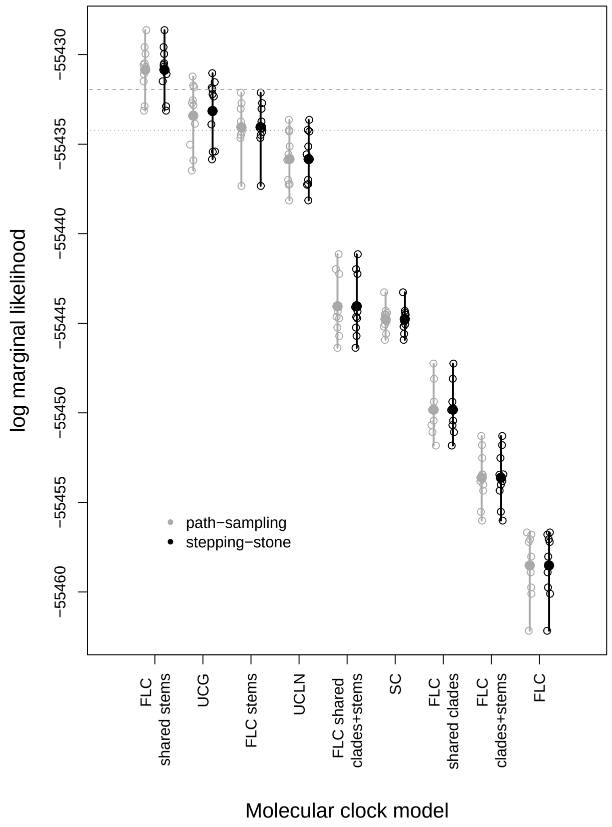

**FIG. 1.** Calculations of log marginal likelihoods for all molecular clock models using path-sampling and stepping-stone. The hollow circles represent individual estimates, with 10 replicates per model, and solid circles denote the mean value over replicates. The vertical lines represent the range of values in each case. The horizontal dashed line corresponds to a log Bayes factor of 1.1 ('substantial evidence') relative to the mean log marginal likelihood of the best model (FLC shared stems), while the dotted line is the same value relative to the lowest log marginal likelihood of the best model.

of the posterior density was higher than the mean background rate (Fig 2).

A key consideration is that the high uncertainty in the FLC stems model means that the actual values of rate estimates for VOC stem branches should be interpreted cautiously. The prior on all clock rates here is a continuous-time Markov chain (CTMC) reference prior, which consists of a $\Gamma$ distribution with $\alpha$=0.5 and $\beta$=T, where T is the tree length (Ferreira and Suchard, 2008; Wang and Yang, 2014). Because the mean of a $\Gamma$ distribution is $\alpha/\beta$, under this prior the expectation is that the average substitution rate is 0.5/T. Our estimate of T under this model had a mean of 85.6 (95% CI: 78.37 - 92.98), which results in a relatively wide distribution with an expected mean of around $5\times10^{-3}$ subs/site/year. A comparison of this prior, the posterior for VOC stem branch rates, and the background rate illustrates that VOC branch rates deviate much less from the prior than the background rate does. Thus, VOC branch rate estimates under this model may be sensitive to the choice of prior (see Supplementary material, Figs S2 and S3). That is, the data may not be sufficiently informative to produce meaningful estimates of these parameters under this model.

The coefficient of rate variation for both relaxed clock models, UCG and UCLN, was indicative of departure from clocklike evolution in the data. To investigate whether VOC stem branch rates differed from the rest, we extracted individual branch rates and compared the VOC stem branch rates to the mean of all other branches. We found evidence that VOC stem branch rates were higher than the mean of other branches, with higher means values, but very high uncertainty and 95% credible intervals that overlapped with the mean of other branches (Fig 3).

The mean substitution rate of branches other than the VOC stems was $0.65\times10^{-3}$ subs/site/year (95% CI: 0.58 - $0.77\times10^{-3}$) in the UCLN and 0.69 $\times10^{-3}$ subs/site/year (95% CI: 0.60 - $0.80\times10^{-3}$)

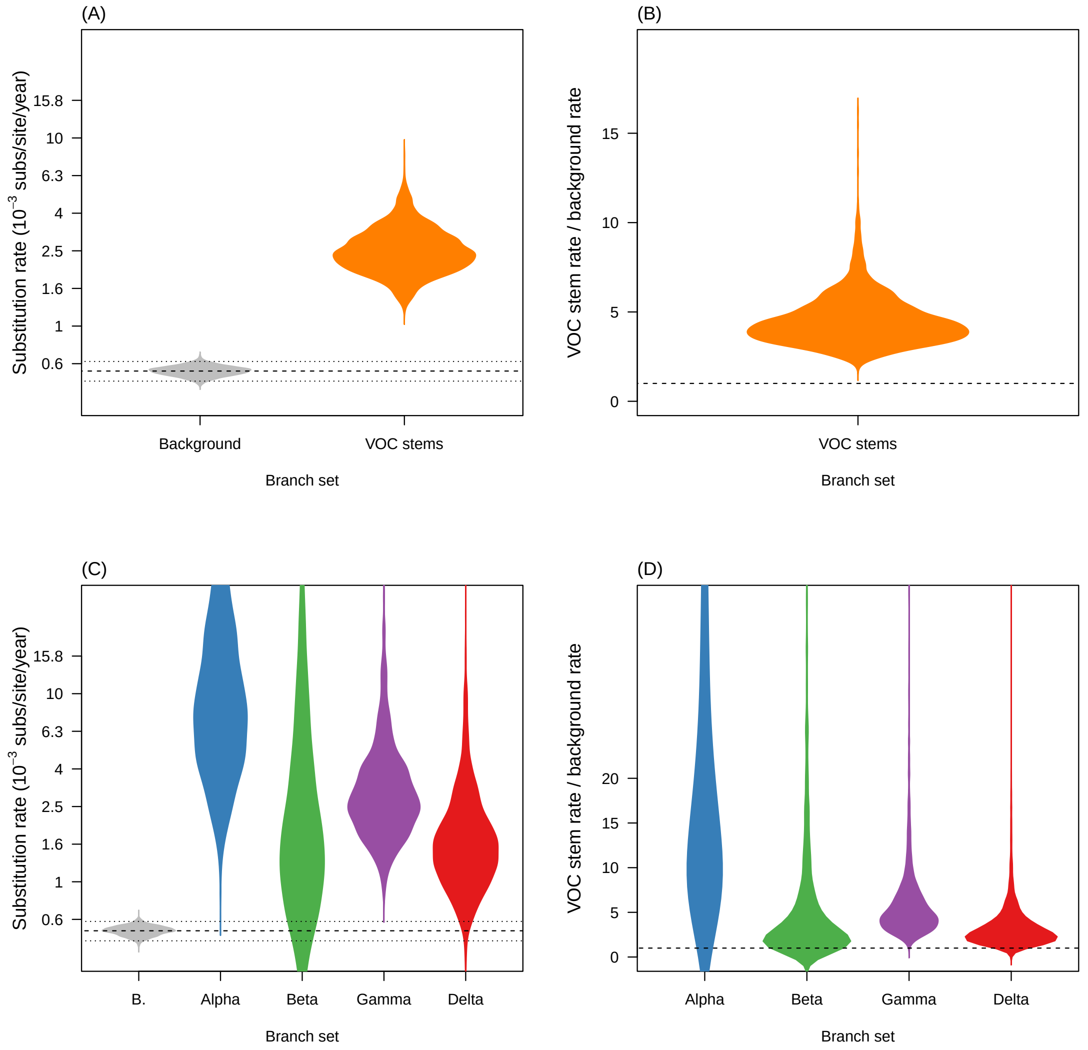

**FIG. 2.** Violin plots for posterior statistics of fixed local clock models (FLC). (A) is for a model where the stem branches of VOCs share a substitution rate that is different to that of the background (model 'FLC shared stems' in Supplementary material Table S1 and Fig S1. The substitution rate for variants of concern (VOC) stem branches is shown in orange and the background in grey. The dashed line represents the mean background rate and the dotted lines are the 95% credible interval. (B) is the ratio of the substitution rate for VOC stem branches and the background under the same model and the dashed line represents a value of 1.0 where the background and VOC stem rate would be the same. (C) and (D) show the corresponding statistics for the FLC stems model, where the stem branch of every VOC has a different rate. Abbreviation 'B.' stands for background.

for the UCG. In contrast, the VOC stem mean substitution rates for the UCLN were: $1.29\times10^{-3}$ subs/site/year (95% credible interval, CI: 0.76 - $2.56\times10^{-3}$) for Alpha , $0.64\times10^{-3}$ (95% CI: 0.32 - $1.57\times10^{-3}$) for Beta, $1.29\times10^{-3}$ (0.82 - $2.40\times10^{-3}$) for Gamma, and $1.06\times10^{-3}$ (95% CI: 0.50 - $2.38\times10^{-3}$) for Delta, and with comparable values for the UCG. The quantile where VOC stems rates fell with respect to other branches also supported the finding that their rates were particularly high in most cases. In the UCLN, for Alpha 0.96 of posterior density had the stem rate in the top 75% of fastest evolving branches, with the corresponding numbers for the other VOCs being 0.25, 0.98, and 0.81 Beta, Gamma, and

Delta, respectively, and with comparable values in the UCG (0.92, 0.45, 0.96, and 0.91; Fig 3).

The RLC model produced less clear results than the other molecular clock models. The maximum *a posteriori* number of rate changes was 4, with the 95% CI ranging between 2 and 5. However, the posterior probability of rate changes in VOC stem branches or clades was 0.0. Instead, rate changes were not consistently found on particular branches. It is conceivable that this model poses a heavy penalty on rate changes. In particular, there is a very large number of local clock configurations in these data, which may be impossible to visit under a standard Markov chain Monte Carlo analyses and may result in low statistical power to assess support for our hypotheses. This model had a substitution rate estimate that was comparable to that of other models (mean $0.60\times10^{-3}$ subs/site/year; 95% CI: 0.49 - $0.72\times10^{-3}$).

### Emergence time and expected genome substitutions

We estimated the duration of time along VOC stem branches and the inferred total number of nucleotide substitutions along the complete genome. We focus on the best fitting model (FLC shared stems), with similar results for other models (Supplementary material Fig S4). The duration of time along these branches represents the time required before VOCs started to diversify, but it is important to note that they are contingent on sampling bias, and could therefore be shorter than estimated here. Under the FLC shared stems model, the stem branch leading up to VOs were; 14 weeks (95% CI:6 - 24) for Alpha, 4 (95% CI: 2 - 8) for Beta, 17 (95% CI: 8 - 28) for Gamma, and 6 (3 - 11) for Delta (Supplementary material Fig S4).

The expected number of substitutions along the complete genome were; 21 (95% CI: 14 - 32) for Alpha, 6 (95% CI:3 - 11) for Beta, 26 (95% CI: 18 - 35) for Gamma, and 9 (95% CI: 6 - 16) for Delta. Although, these numbers are loosely associated with the defining mutations, they are not directly comparable because they involve substitutions along the entire genome and they correspond to the inference from a standard phylogenetic substitution model (the GTR+$\Gamma$ in this case).

## Discussion

Our mean rate estimates over all lineages are somewhat lower than earlier estimates (Duchene *et al.*, 2020), which is consistent with the notion that the virus has had time to evolve and to remove transient deleterious mutations since its emergence (Ghafari *et al.*, 2021). Clearly, the molecular substitution rate of SARS-CoV-2 displays substantial variation among lineages, a pattern that has been apparent since early phylogenetic analyses of the virus (Duchene *et al.*, 2020).

Substitution rate variation is sometimes stochastic in nature and pinpointing its causes is often difficult in empirical data. Our explicit

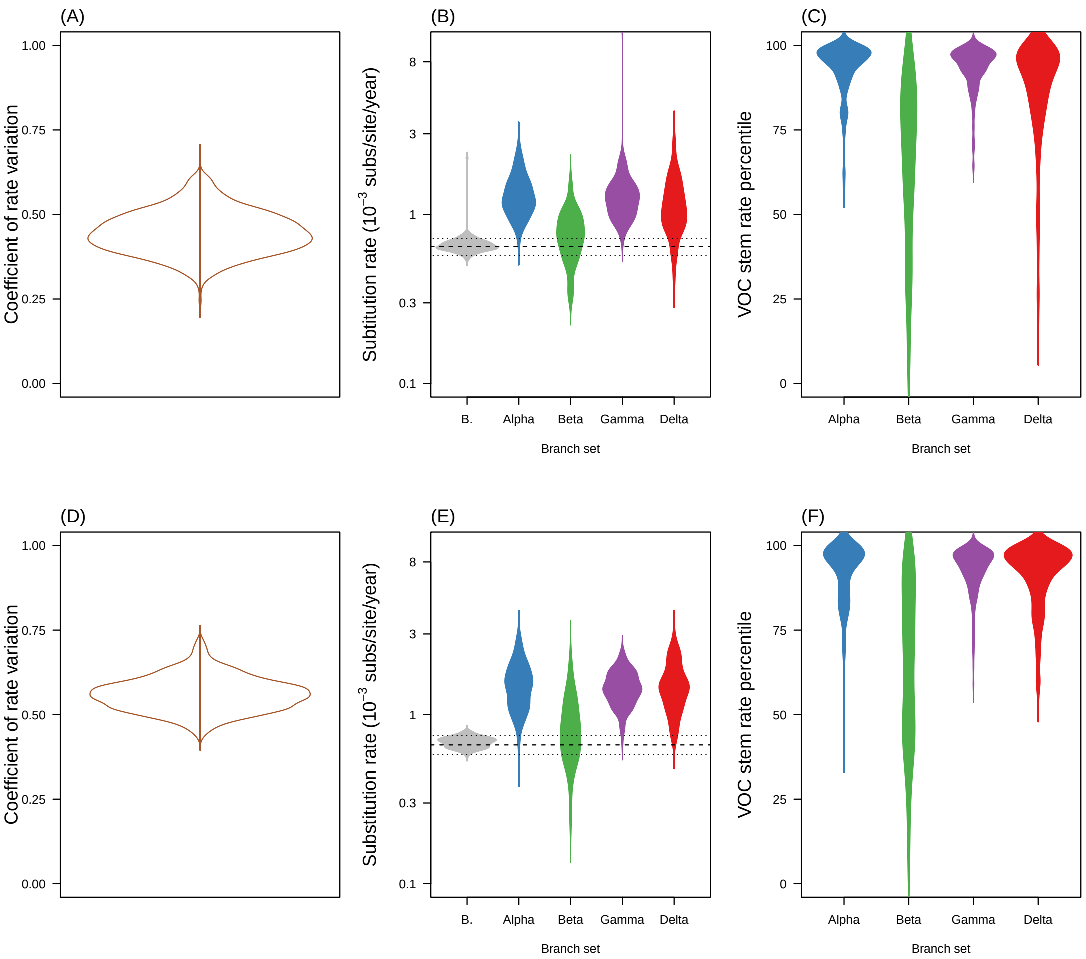

**FIG. 3.** Violin plots of posterior statistics for the uncorrelated relaxed clocks with lognormal (UCLN) and gamma (UCG) distributions (see Supplementary material). The top row, (A) through (C), is for the UCLN and the bottom row, (D) through (F), is for the UCG. (A) and (D) show the coefficient of rate variation, which is the standard deviation of branch rates divided by the mean rate, and indicates clocklike behaviour when it is abutting zero (Drummond *et al.*, 2006; Ho *et al.*, 2015). In (B) and (E) the substitution rate is shown for the stem branches of variants of concern (VOCs) and for the mean of background branches (i.e. those that are not the stems of VOCs), abbreviated as 'B.'. The dashed line denotes the mean background rate, while the dotted lines represent the upper and lower 95% credible interval. (C) and (F) shows the percentile in which stem branches for VOCs fall with respect to other branches. Note that the densities have been smoothed, but the maximum values are 100.

hypothesis testing framework suggest that the emergence of VOCs explains much of the substitution rate variation in the virus. This model testing framework has been previously used to understand viral evolution among host species in influenza (Worobey *et al.*, 2014), and the host range SARS-CoV-2 and closely related viruses (MacLean *et al.*, 2021). Here we used marginal likelihood estimators that have shown high accuracy (Fourment *et al.*, 2020), but recent developments, including those based in sequential Monte Carlo (Wang *et al.*, 2020), may improve statistical power for differentiating clock models. We suggest that model testing may be preferable to using highly parametric models, such as relaxed molecular clock models, for this purpose because they tend to have very high variance in substitution rates of particular branches. Recent advances in fitting relaxed and random local clock models may provide increased sensitivity and precision in branch specific rate estimates (Douglas *et al.*, 2021; Fisher *et al.*, 2021) .

We find compelling evidence that episodic, instead of long-term, increases in the substitution rate underpin the emergence of VOCs, a process that is probably driven the action of natural selection. All models where VOC clades were assigned a different rate to the background had poor statistical fit, even when compared to the SC 'null' model, providing further support for such rate increases to occur over a short period of time. The increase in substitution rate required to give rise to the four VOCs examined was estimated to be around 4-fold compared to the background, although such estimates may carry high uncertainty when estimated for individual stem branches. Under these circumstances the number of mutations required to give rise to a VOC, such as Alpha, would have accumulated in about three months, with some variants requiring a few weeks, such as Beta and Delta. These timescales appear plausible in chronic infections of SARS-CoV-2 (Harvey *et al.*, 2021; Kemp *et al.*, 2021), but other circumstances are also likely, for example if transmission is infrequent and selection favours mutations that increase transmissibility between hosts.

Our genomic analyses demonstrate that signatures of increased substitution rates are detectable using phylogenetic methods and genome surveillance data. A recent study of reported increased evolutionary rates within sublineages of Gamma (Gräf *et al.*, 2021), which prompts further investigation of within lineage evolution. However, the precise mechanism (ecological or intrahost) of how VOCs have emerged is still unclear. Elucidating these processes will require dense sampling between transmission chains, specifically in settings where transmission is unlikely and intra-host sequence data is available. An other important area that is currently under intense investigation is how natural selection shapes the emergence and persistence of VOCs (Martin *et al.*, 2021; Tegally *et al.*, 2021). Such studies may benefit from using explicit models where the substitution rate is treated as a function of environmental or ecological variables (Streicker *et al.*, 2012). We recommend that further research focuses on early detection and understanding of the circumstances under which viral lineages with epidemiological impacts, such as VOCs, emerge.

## Materials and methods

### Data set construction

We downloaded 100 randomly selected sequences in the global NextStrain SARS-CoV-2 build of August 2021 (Hadfield *et al.*, 2018), from the GISAID database (Elbe and Buckland-Merrett, 2017; Shu and McCauley, 2017). This set of sequences did not include any of those belonging to the four VOCs (Alpha, Beta, Gamma, or Delta) and we also excluded samples drawn from non human hosts. We then downloaded 20 randomly selected sequences from the four

VOCs to generate a data set of 180 genomes, which we aligned using MAFFT (Katoh and Standley, 2013). We ensured that the sequences consisted of complete genomes, with no stretches of more than 10 Ns and excluding those with low coverage (see Supplementary material). To verify that samples classified as VOCs were correctly assigned as such, we estimated a phylogenetic tree using maximum likelihood as implemented in IQ-TREE2 (Minh *et al.*, 2020), using the GTR+Γ substitution model and with approximate Bayes branch support (Anisimova *et al.*, 2011). We ensured that all VOCs were monophyletic with an approximate Bayes support of at least <0.95.

### Bayesian phylogenetic analyses

Our Bayesian analyses require specifying a substitution model, a tree prior and priors for all parameters in BEAST 1.10 (Suchard *et al.*, 2018). We chose the GTR+$\Gamma_4$ substitution model and a coalescent exponential tree prior. Although the tree prior is not necessarily realistic here, it is expected to have little impact in molecular clock estimates (Ritchie *et al.*, 2017). It can accommodate changes in population size via the exponential growth function and it is fully parametric, meaning that setting proper priors for all parameters is possible. To calibrate the molecular clock we specified the sequence sampling times. The FLC models require constraining monophyly of VOCs, which we also did for other clock models to ensure that the prior on tree topology was the same.

We used the default priors for the substitution model. The coalescent exponential tree prior has two parameters, the scaled population size, $\Phi$, and the growth rate $r$. The scaled population size is proportional to the number of infected individuals at present divided by the twice the coalescent rate, $\lambda$, (i.e. $\Phi = \frac{I(0)}{2\lambda}$) and the growth rate is inversely proportional to the doubling time by a factor of log(2) ($doubling\ time = \frac{log(2)}{r}$) (Boskova *et al.*, 2014; Volz *et al.*, 2009). We used priors with relatively low information content for these two parameters. For $\Phi$ we used an exponential distribution with mean $10^5$, while for $r$ we used a Laplace distribution with location 0 and scale 100. For all molecular clock rates we used a CTMC reference prior (Ferreira and Suchard, 2008; Wang and Yang, 2014). The UCLN and UCG models have an additional parameter; the standard deviation of the lognormal distribution, and the shape of the Γ distribution. For these parameters we specified an exponential prior with mean 0.33. We ran our analyses for using a Markov chain Monte Carlo of length $5\times10^7$, sampling every $5\times10^3$ and discarding 10% of the chain as burn-in. We repeated the analyses once to verify convergence of independent chains and we ensured that the effective sample size of all parameters was at least 200.

### Marginal likelihood estimation

We used two techniques to infer the log marginal likelihood; path-sampling and stepping-stone (Gelman and Meng, 1998; Lartillot and

Philippe, 2006; Xie *et al.*, 2011), which have been found to have high performance in differentiating models in phylogenetics (Baele *et al.*, 2012, 2013; Fourment *et al.*, 2020), reviewed by Baele and Lemey (2014); Oaks *et al.* (2019). We chose these estimators over the more recently developed and highly accurate generalized stepping-stone because the latter requires a working genealogical distribution (Baele *et al.*, 2016), which is not trivial here due to the monophyletic constraints in our models. Our estimation setup had 200 path steps distributed according to quantiles from a $\beta$ distribution with parameter $\alpha$=0.3, with each of the resulting 201 power posterior inferences running for $10^6$ iterations. We repeated these analyses ten times to assess variation in these calculations and used the log Bayes factors of the mean values. Our model testing approach considered the UCLN, SC, and all FLC models in Table 1 and Supplementary material. We did not calculate log marginal likelihoods for the RLC because this is a model averaging method, where the number of parameters is less tractable than in other models and thus it is difficult to conceive proper priors for all parameters, a fundamental aspect of Bayesian model selection.

## Supplementary Material

Supplementary material available at Molecular Biology and Evolution online (http://www.mbe.oxfordjournals.org/).

## Acknowledgments

This work was supported by the Australian Research Council (DE190100805) and the Medical Research Future Fund (MRF9200006). This research was undertaken using the LIEF HPC-GPGPU Facility hosted at the University of Melbourne. This Facility was established with the assistance of LIEF Grant LE170100200. We acknowledge efforts by originating and submitting laboratories for the sequence data in GISAID EpiCoV on which our analyses are based. We are grateful to Prof. Edward Holmes for useful suggestions and comments on ideas developed in this study. We also thank two anonymous reviewers for useful suggestion and comments of earlier versions of this manuscript.

## Supplementary Material

msac013_Supplementary_Data

