# Supplementary material for: The Emergence of SARS-CoV-2 Variants of Concern Is Driven by Acceleration of the Substitution Rate
Source: Mol Biol Evol. 2022 Jan 17;39(2):msac013. doi: 10.1093/molbev/msac013 (PMC8807201; doi:10.1093/molbev/msac013)
Supplement: msac013_Supplementary_Data [file msac013_supplementary_data.zip › suppInfo.pdf]

The emergence of SARS-CoV-2 variants of concern is driven by acceleration of the substitution rate

John H. Tay, Ashleigh F Porter, Wytamma Wirth, Sebastian Duchene*.

1 Supplementary material

GISAID acknowledgements table

Table S1: Molecular clock model configurations and parameterisation. The term 'clades' correspond to monophyletic groups of either of the four variants of concern (VOC) in the data set (Alpha, Beta, Gamma, Delta) and the stems are the branches leading up to them. Note that 'clock rate' refers to the global clock rates of the strict clock and 'b. rate' is for the background rate.

Model family	Model abbreviation	Parameters	Number of parameters
Strict clock	SC	clock rate	1
Relaxed	UCLN	Num. branches, mean, s.d.	358+2
Relaxed	UGM	Num. branches, mean, shape	358+2
Random local clock	RLC*	b. rate, num. rate changes	variable
Fixed local clock	FLC clades	b. rate, num. clades	1+4
Fixed local clock	FLC stems+clades	b. rate, num. clades & stems	1+4
Fixed local clock	FLC stems only	b. rate, num. stems	1+4
Fixed local clock	FLC shared stem	b. rate, stem rate	1+1
Fixed local clock	FLC shared clade	b. rate, clade rate	1+1
Fixed local clock	FLC shared clades+stems	b. rate, clade & stem rate	1+1

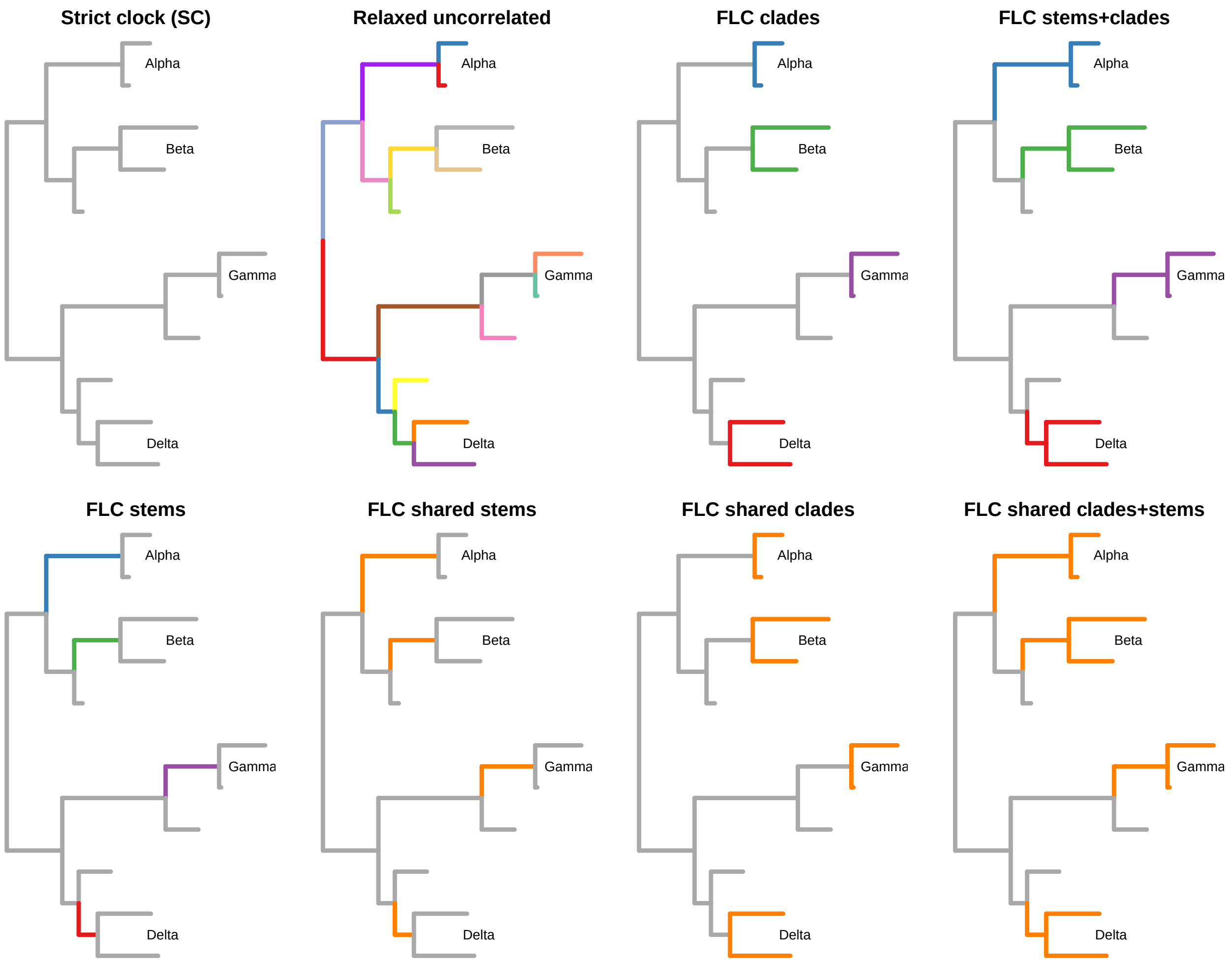

Figure S1: Illustration of molecular clock models considered in hypothetical trees with the four variants of concern (VOCs) and background genetic diversity. SC stands for strict clock and FLC for fixed local clock. Branches are coloured according to the rate assigned in each case, with grey corresponding to the 'background' branches and those in colours being the 'foreground' branches. Model names match those of Supplementary material Table 1.

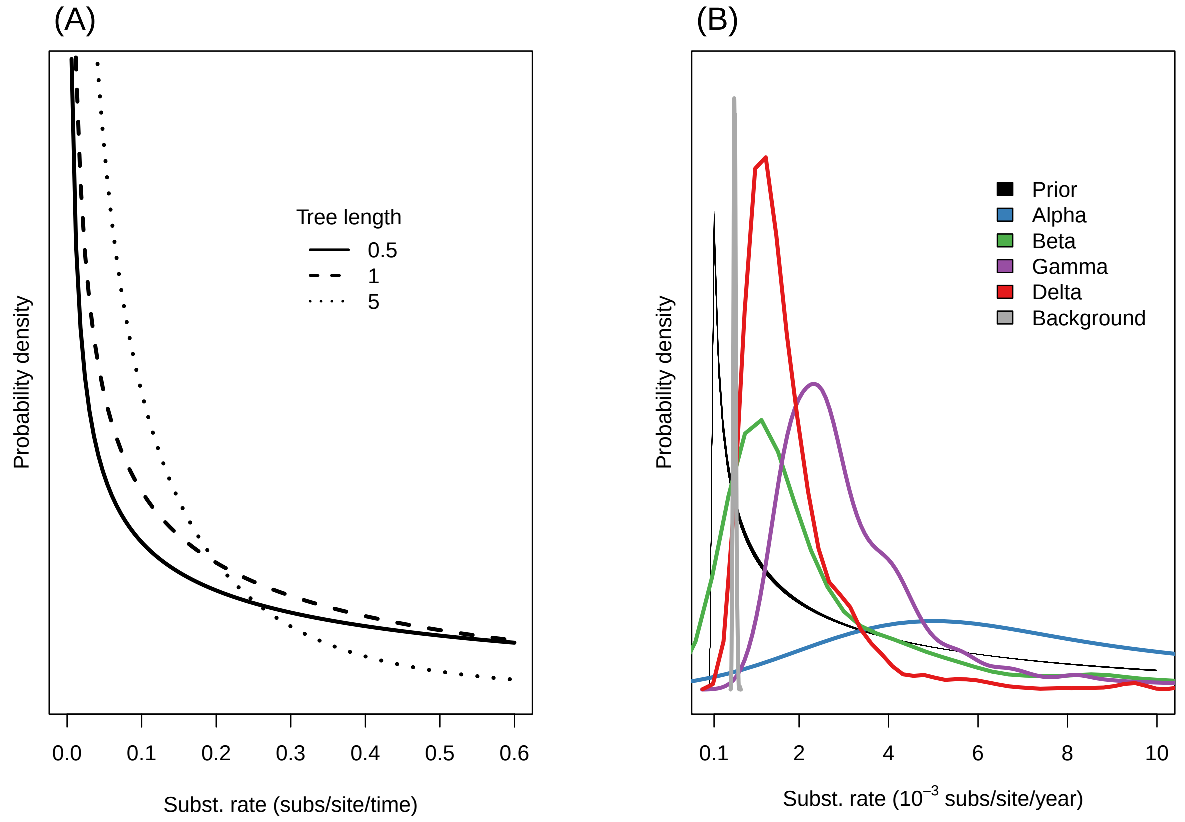

Figure S2: Panel (A) is an illustration of the continuous time Markov chain (CTMC) reference prior (Ferreira and Suchard 2008; see also Wang et al. 2014), defined as a Γ distribution with α=0.5 and β=T and where T is the tree length. As the tree length increases, the prior density is more concentrated on lower clock rate values. Panel (B) shows 100 realisations of the CTMC prior by sampling values from the posterior distribution from the FLC stems model and plotting individual lines (because the tree length has a narrow posterior the lines are overlaid on top of each other, that is, the lines are very similar). The posterior estimates for the VOC stem branches and that for the background branches are shown. Note that the VOC stem branch rate estimates are much wider than that for the background and may therefore be more sensitive to the prior.

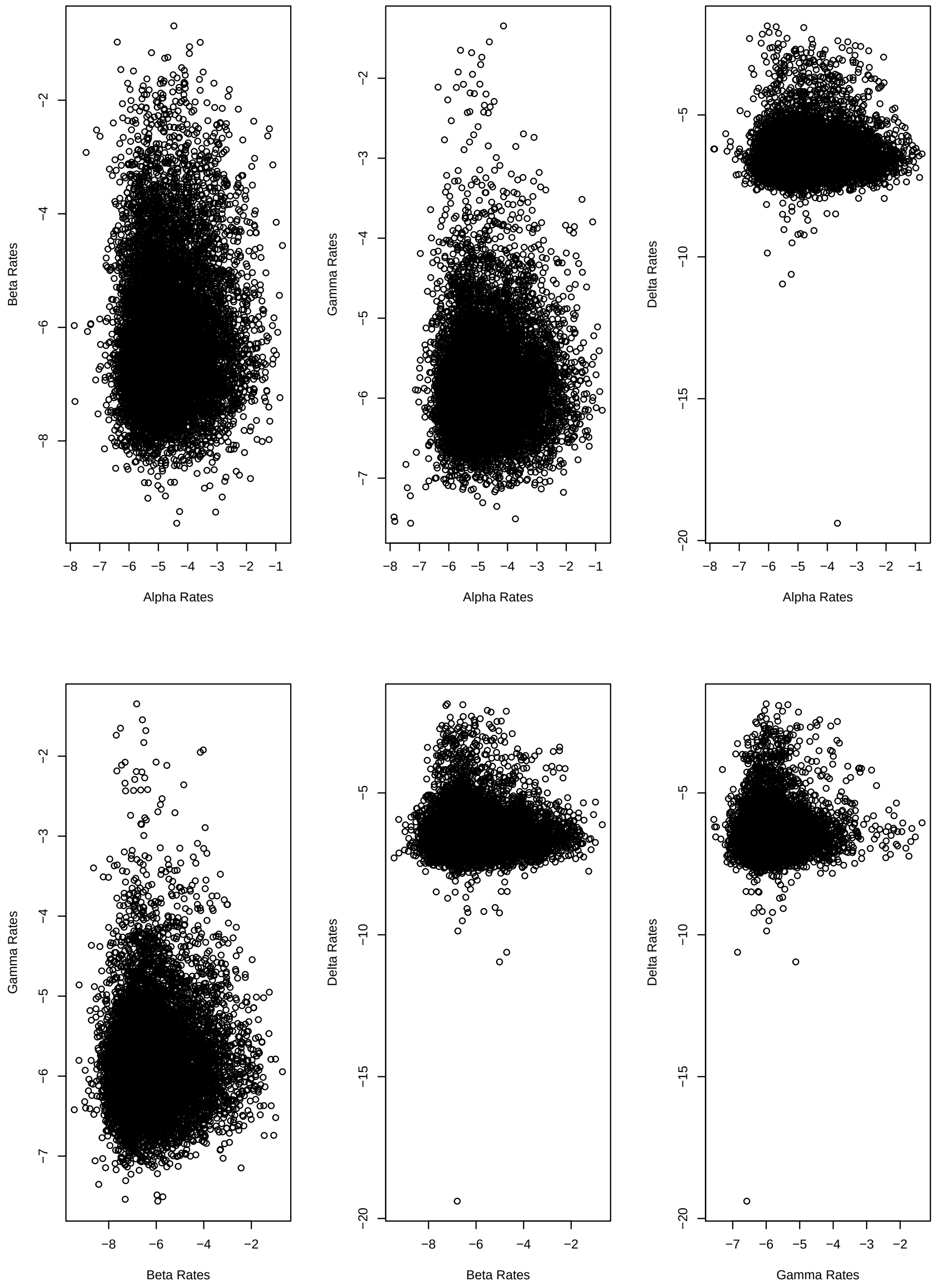

Figure S3: Pairwise plots between VOC stem branch rate estimates under the FLC stems model. Each axis denotes the log transformed rate for each VOC and points are draws from the posterior distribution. Note that there is no obvious correlation between any pair of VOC branch rates, which is sometimes a symptom of overparameterisation, and does not seem to affect these analyses.

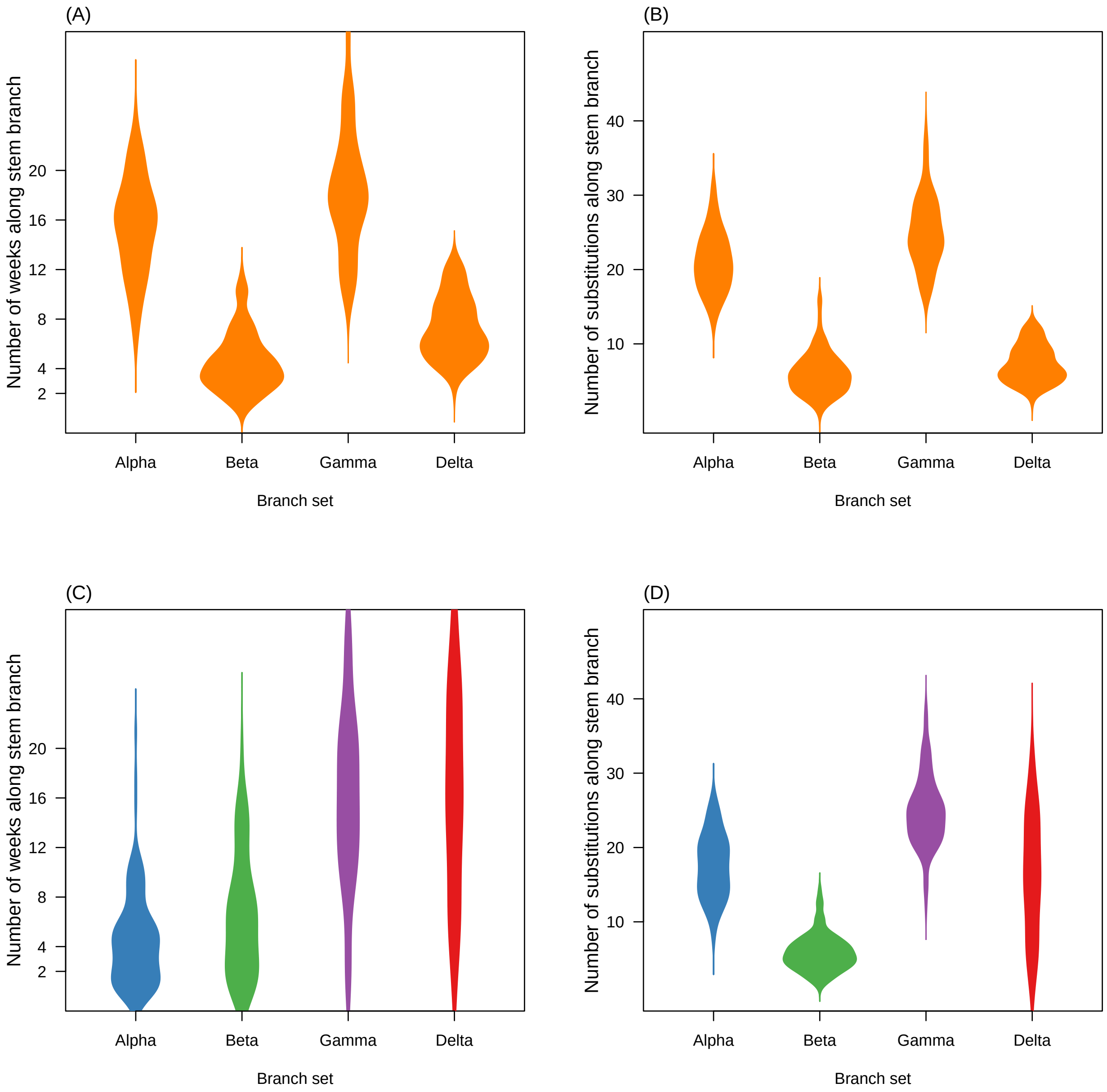

Figure S4: Violin plots of the number of weeks and expected number of substitutions along VOC stem branches for the FLC shared stems model (panels (A) and (B)) and FLC stems (panels (C) and (D)).
